# Synthesis and characterization of ultralong SiC nanowires with unique optical properties, excellent thermal stability and flexible nanomechanical properties

**DOI:** 10.1038/s41598-017-03588-x

**Published:** 2017-06-07

**Authors:** Ping Hu, Shun Dong, Xinghong Zhang, Kaixuan Gui, Guiqing Chen, Ze Hu

**Affiliations:** 1 Science and Technology on Advanced Composites in Special Environment Laboratory, Harbin Institute of Technology, Harbin, 150001 P.R. China; 2 School of Computer Science and Technology, Harbin Institute of Technology, Harbin, 150001 P.R. China

## Abstract

Several-millimeter long SiC nanowires (NWs) with unique optical properties, excellent thermal stability and flexible nanomechanical properties were synthesized using a simple method with silicon and phenolic resin as the raw materials. The SiC NWs displayed special optical properties that were attributed to their large size and Al-doping. They displayed broad green emission at 527.8 nm (2.35 eV) and purple emission concentrated at 438.9 nm (2.83 eV), in contrast to the other results, and the synthesized SiC NWs could also remain relatively stable in air up to 1000 °C indicating excellent thermal stability. The Young’s moduli of the SiC NWs with a wide range of NW diameters (215–400 nm) were measured using an *in situ* nanoindentation method with a hybrid scanning electron microscopy/scanning probe microscopy (SEM/SPM) system for the first time. The results suggested that the values of the Young’s modulus of the SiC NWs showed no clear size dependence, and the corresponding Young’s moduli of the SiC NWs with diameters of 215 nm, 320 nm, and 400 nm were approximately 559.1 GPa, 540.0 GPa and 576.5 GPa, respectively. These findings provide value and guidance for studying and understanding the properties of SiC nanomaterials and for expanding their possible applications.

## Introduction

Recently, SiC NWs, as one of the most promising nanoscale building blocks, have attracted increasing interest due to their superior mechanical properties^1^, high temperature oxidation resistance^2^, excellent chemical stability^3^, low thermal expansion coefficients^4^, high thermal conductivity^5^, and large band gap^5^, which enables their applications in composite reinforcements^6, 7^, nanodevices and optoelectronics^1, 3^. For example, Yang *et al*. significantly improved the mechanical properties of SiC/SiC composites by the *in situ* growth of SiC NWs with a low volume fraction of 6.1%, which displayed an approximately doubled fracture toughness and 97% increased strength^7^. Zhang and co-workers reported that the introduction of *in situ* SiC NWs led to an increase in the bonding strength of a Si-Mo-Cr coating by 5.7%, and the mass loss per unit area was reduced by 46.39% after 30 thermal cycles between 1600 °C and room temperature^8^. The above results showed the effective reinforcement efficiency of SiC NWs in terms of mechanical properties. Therefore, an accurate method for measuring the mechanical properties of SiC NWs is of critical importance before integrating them into functional composites and nanodevices since mechanical failure may result in the failure or malfunction of the composites and nanodevices^9, 10^.

To date, various techniques for measuring the mechanical behaviors and properties of individual NWs have been developed, such as *in situ* bending tests^11^, tensile tests^12–15^, resonant frequency tests^16^ and nanoindentation^17^, and investigations on the mechanical properties of the NWs have been reported in the literature. Lieber *et al*. first reported the Young’s moduli and fracture strengths of SiC NWs using a bending test with atomic force microscopy (AFM), showing the Young’s moduli of the SiC NWs to be 610 GPa and 660 GPa for 23 nm and 21.5 nm diameter NWs, respectively^18^. Cheng *et al*. conducted a quantitative mechanical characterization of SiC NWs with diameters in the range of 17–45 nm via an *in situ* tensile test inside an SEM, in which the Young’s moduli exhibited a large range from 166 to 1270 GPa, with an average value of 531 GPa^10^. Perisanu *et al*. measured the mechanical properties of different diameters of SiC NWs that ranged from 17.5 to 143 nm, as measured by SEM and the field emission (FE) from the mechanical resonances of single-clamped NWs, and the Young’s moduli ranged from 230 to 750 GPa^19^. Meanwhile, molecular-dynamics methods have also been employed to calculate the mechanical properties of SiC NWs with of several nanometers in diameter^20–23^. Previous studies have mainly concentrated on the mechanical properties of SiC NWs of no more than 300 nm in diameter or SiC whiskers^24, 25^. In addition, the Young’s moduli of SiC NWs calculated from nanoindentation measurements have not been reported until now, according to our survey.

Here, we report our successive work on the synthesis of ultralong SiC NWs using an effective method, in which the optical and thermal stability properties of the SiC NWs were explored. Meanwhile, the mechanical properties of the SiC NWs with different diameters were also measured via *in situ* nanoindentation tests conducted with a hybrid SEM/SPM system, for the first time, to further explore their potential applications. Moreover, the experimental data cover a wide range of NW diameters (215–400 nm) used in this research field, to our best knowledge, and thus, we expect that the evaluated NW mechanical properties will be valuable for a better understanding of NW mechanics.

## Results and Discussion

### Characterization and growth mechanism of the ultralong SiC NWs

**Figure 1:**
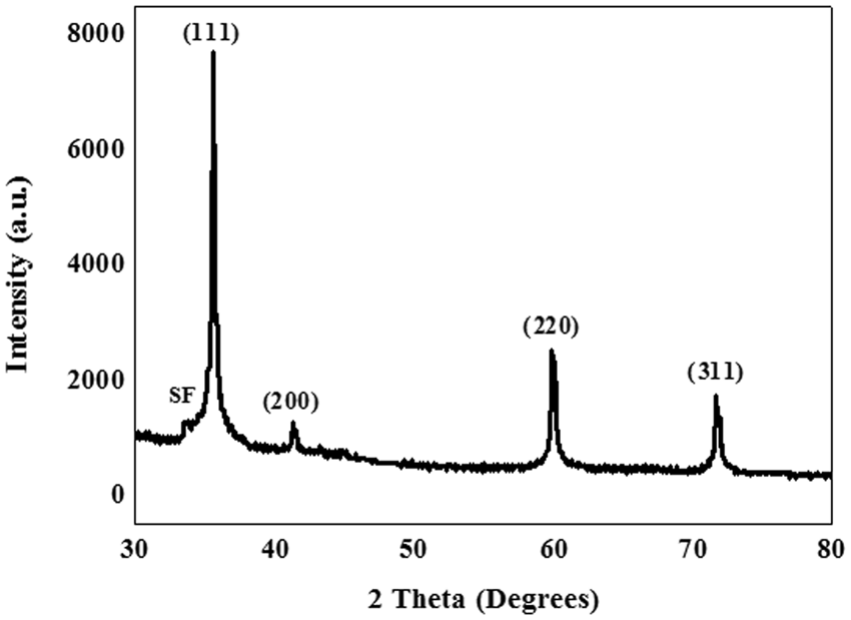
XRD patterns of the white, wool-like, products obtained from the inner wall of the ceramic crucible.

**Figure 2:**
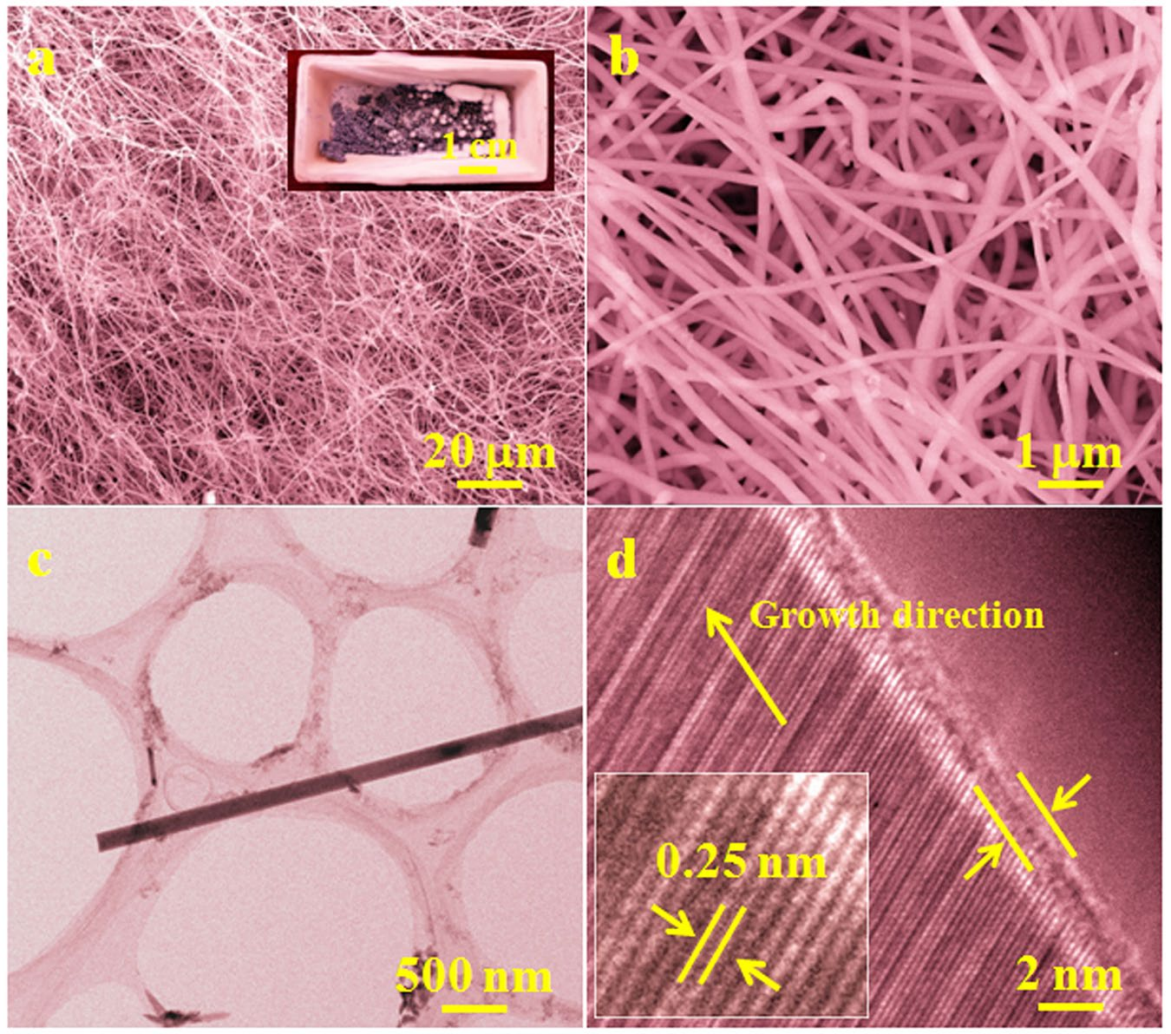
(**a**,**b**) SEM and (**c**,**d**) TEM images of the white, wool-like, products obtained from the inner wall of the ceramic crucible. The inset image in (**d**) shows the partial enlarged image of (**d**).

**Figure 3:**
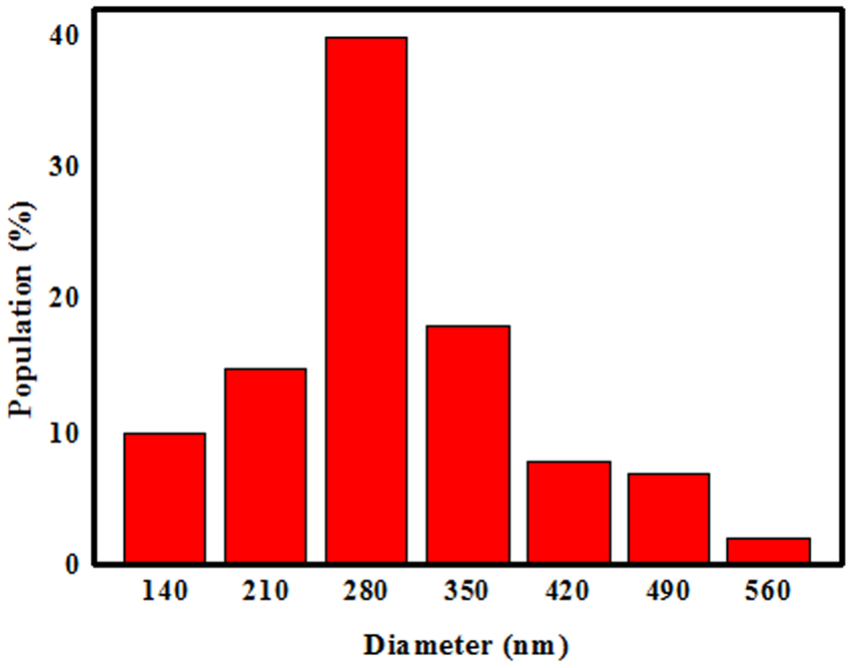
The width distribution histogram of the white, wool-like, products obtained from the inner wall of the ceramic crucible.

The crystalline phases of the products grown on the surfaces of the inner walls of an alumina crucible, at 1450 °C with a rate of 50 ml/min of Ar, were characterized using large-scale XRD. From the XRD patterns shown in Fig. 1, the positions and intensities of these diffraction peaks were in good agreement with the standard values of β-SiC, in which the major strong peaks were attributed to the (111), (200), (220), and (311) lattice planes of β-SiC, respectively. A low-intensity peak at a lower diffraction angle than that of the strong (111) peak was marked as SF, which was ascribed to the presence of stacking faults^26–28^. The morphologies and microstructures of the synthesized white wool-like products obtained on the inner walls of the alumina crucible were examined using SEM, as shown in Fig. 2a,b. From the macroscopic morphology shown in the inset of Fig. 2a, it can be seen that the lengths of the white wool-like products grown on the surface of the mixture powder and inner wall of the alumina crucible were as high as several millimeters. The products exhibited both straight and curved shapes with lengths of several hundred micrometers, and the diameters of the nanomaterials were not uniform. To further analyze the microstructures of the SiC NWs, TEM and HRTEM were employed, and the results are displayed in Fig. 2c,d. From the TEM images, the diameter of the NWs was found to be approximately 300 nm, and the NWs were observed to possess a uniform diameter along their entire length with a clean surface. From the typical HRTEM image in Fig. 2d, the SiC NWs were composed of a crystalline core coated with an approximately 2 nm thick, thin amorphous, shell layer, and a few stacking faults were also observed along the growth direction of the SiC NWs. The insert in Fig. 2d shows that the inter-planar spacing perpendicular to the SiC NW axis was approximately 0.25 nm, which is consistent with the (111) plane spaceing of β-SiC, suggesting that the growth direction of the SiC NW occurred along [111]^29–31^. Meanwhile, a diameter distribution histogram of the NWs from the statistical measurements based on the SEM images indicated an average diameter of 285 nm with the main distribution ranging from 210 to 350 nm, as shown in Fig. 3. Therefore, it was reasonable to believe that this simple method might be an effective way to prepare ultralong SiC NWs.

According to the results in the literature, the growth mechanism of the white, wool-like, ultralong, SiC NWs was attributed to a typical vapor-solid (VS) growth model. This theory was supported by the lack of droplets found and by the lack of a catalyst^32–35^. The growth process of the SiC NWs was concluded to occur in three stages: incubation, nucleation and growth^36–40^. First, large amounts of gasses were generated due to the decomposition of the phenolic resin, including CO, CH_4_ and H_2_, in the early stages of the heating process^40–42^. Some reactions would occur in sequence to produce CO, SiO, and CO_2_ and then form a suitable atmosphere for nucleation when the temperature increased^40–42^. Second, reactions could occur under the right conditions to form SiC nuclei when the temperature increased, and the SiC clusters acted as nucleation centers for the catalyst-free VS growth mechanism^40^. However, it should be noted that the SiC clusters might have formed due to the following driving forces. The first includes the anisotropic properties of the different surfaces in the crystal, including the preferential reactivity and binding of the gas reactants on specific surfaces^43^. All crystals tend to minimize their total surface energy, and the wire-like shapes would be formed as the gas reactants were continuously supplied. The second includes the crystal defects, such as stacking faults and twins, and some kink sites were exhibited in the NWs, as shown in Fig. 2b ^43^. Owing to the presence of the kink sites, the crystal or cluster would further grow perpendicularl to the surface due to the addition of atoms, and the growth rate also would be faster than that predicted for a perfect crystal because real crystals contain defects^43^. After the nucleation was formed, the last stage was the subsequent growth of the SiC NWs, which grew subsequently along a fixed axis. The growth direction of the SiC NWs was preferentially normal to the (111) plane of the β-SiC from a crystallographic viewpoint, and SiC NWs would grow subsequently along the fixed direction as long as the gas reactants were continuously produced. Then, large-scale and several-millimeter long SiC NWs were formed^40, 44^. Notably, the pyrolysis process of the phenolic resin played an important role in the formation of the several-millimeter long SiC NWs since only a few such NWs were obtained from the comparison experiment, which was carried out using the raw materials of the resin-derived carbon and silicon; and the other experimental parameters were kept constant.

### Optical and thermal stability properties of the SiC NWs

**Figure 4:**
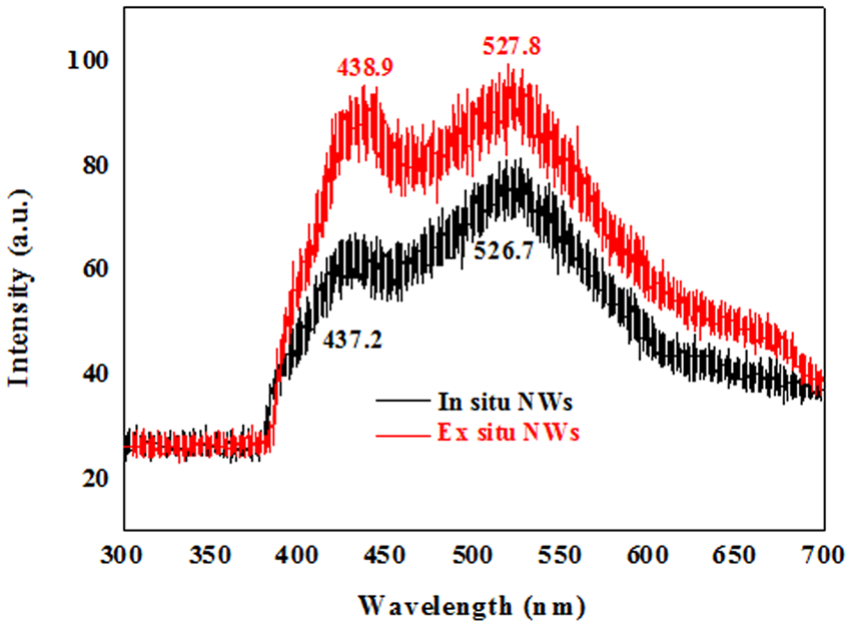
PL spectra of the SiC NWs obtained at different locations under the excitation of a 325 nm He-Cd laser at room temperature.

**Figure 5:**
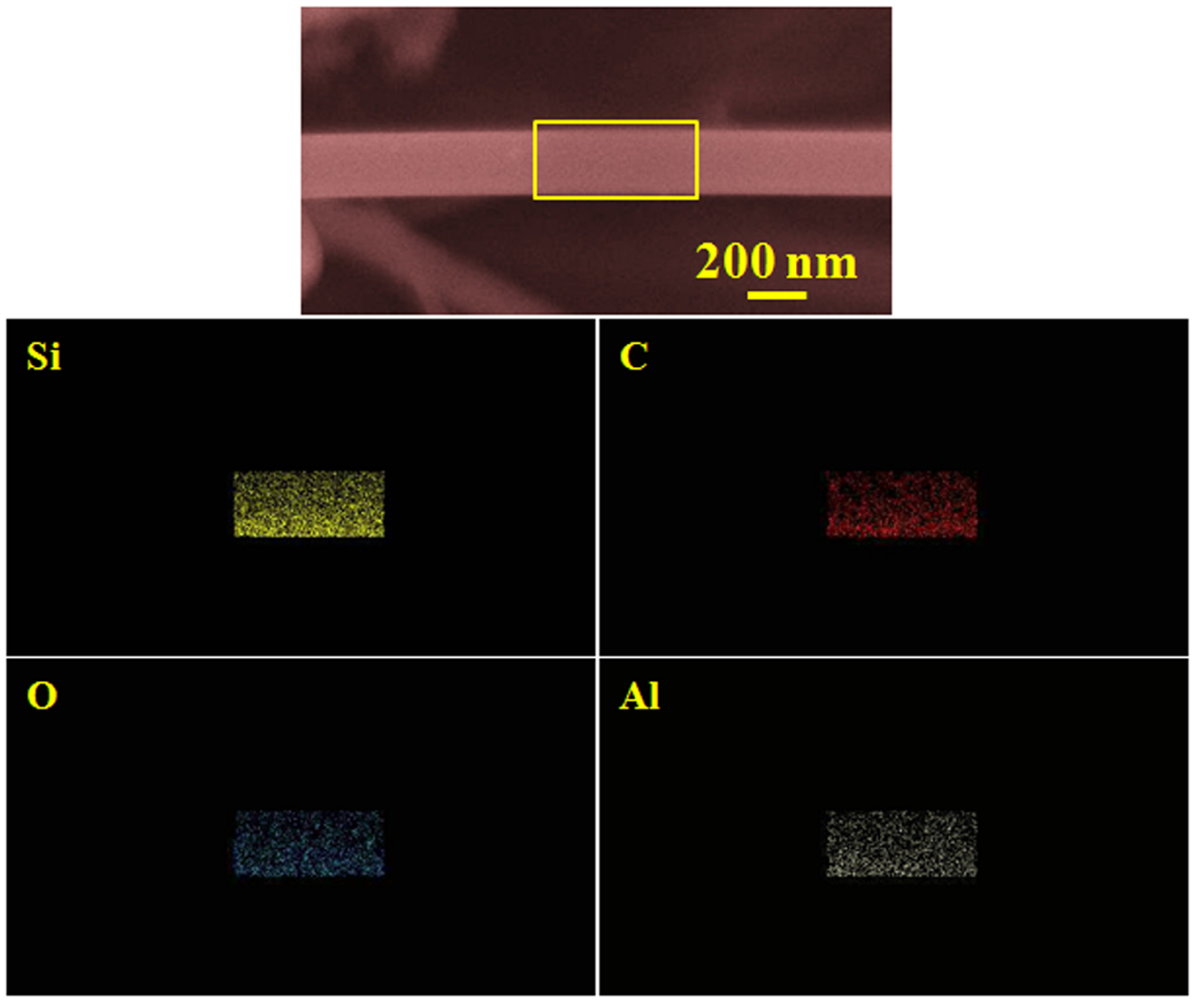
Elemental area scans of the single SiC NWs obtained from the inner wall of the ceramic crucible.

The room temperature PL spectrum of the synthesized white, wool-like, products obtained on the inner wall of the alumina crucible, namely, the *ex situ* NWs, is presented in Fig. 4 with a red line. The spectrum exhibits visible luminescence, giving a broad range emission band between 400 nm and 600 nm, with two obvious peaks at 438.9 nm (2.83 eV) and 527.8 nm (2.35 eV). All of the above peaks showed blueshifts to different extents relative to the bulk 3C-SiC at 539 nm (2.23 eV)^45^. Although, the detailed luminescence mechanism of the NWs is still not fully understood, previous studies have shown that the PL properties of SiC NWs strongly depend on the synthesis conditions, microstructures, and morphologies. The emission peak located at 438 nm was compatible with the values for SiC nanobelts and nanocables, which was attributed to the recombination of quantum confinement and microdefect effects, such as stacking faults in the synthesized SiC NWs^46–48^. However, it was worth noting that the emission peak located at 527.8 nm (2.35 eV) exhibited a redshift compared with most previous results in the literature, while it was similar to the results reported by Gao and co-workers^49^. In addition, the room temperature PL spectrum of the NWs obtained on the surface of the mixture powder, namely, *in situ* NWs, are shown in Fig. 4 with a black line. Compared to the PL spectrum of the *in situ* NWs, the *ex situ* NWs displayed an obvious redshift, and the following reasons can be used to explain this phenomenon. First, we examined the morphology and microstructure of the products grown on the surface of the mixture powder using SEM and TEM along with the diameter distribution histogram of the NWs, as shown in Figs S1 and S2. From the SEM images (Fig. S1a,b), it was observed that the morphologies of the NWs grown on the surface of the mixture powder were similar to the morphologies of the NWs obtained on the inner wall of the alumina crucible, with both straight and curved shapes. Meanwhile, the microstructures and growth directions of the NWs grown at different locations were also similar, as shown in Fig. S1c. However, it is worth noting that the *in situ* NWs had a smaller average diameter of 263 nm compared to that of the *ex situ* NWs with a 285 nm diameter, as shown in Fig. S2. According to previous results in the literature, larger-sized nanostructures could lead to a redshift in the emission spectrum, which could be one of the reasons for the above phenomenon^50^. Second, Gao *et al*. used Al-doping to explain the redshift phenomenon of the emission spectrum, and elemental area scanning was also carried out to study the compositions of a single NW grown at different locations; the results are shown in Figs 5 and S3 ^49^. From Figs 5 and S3, it was observed that the NWs grown at different locations were composed of Si, C and O, while a small amount of Al was found in the NWs obtained from the inner wall of the alumina crucible. Therefore, it was reasonable to believe that the presence of Al contained in the NW grown on the inner wall of the alumina crucible could lead to a redshift in the emission spectrum compared with that of the NW grown on the surface of the mixture powder. The doping mechanism of Al was attributed to the generation of two types of defects with N4^+^ and silicon dangling bonds (K°)^51, 52^. As a consequence, the unique optical properties of these NWs could be attributed to the larger-sized NWs and incorporation of a small amount of Al in the NWs, providing an efficient way to synthesize SiC NWs with desirable optical properties for applications in nanoscale electrical and optoelectronic devices.

In addition, it is worth pointing out that the source of Al should be from the alumina crucible, which also contained alumina and a small amount of Fe_2_O_3_ and SiO_2_ impurities. Fe_2_O_3_ from the crucible could also be reduced to form active Fe nanoclusters under the reductive gas from the reaction system to promote the growth of the NWs^39^. However, no droplets were found at the tips of the NWs according to the SEM and HRTEM images, which could be attributed to the evaporation of Fe-containing compounds in a flowing ambient during the high-temperature process^53^. The alumina from the crucible might have been an effective mediator, playing a significant role in adjusting the concentrations of the reactants containing Si. Based on the previous results reported in the literature, alumina could react with silica to form mullite, and then, the mullite could initiate a liquid-phase separation process to generate liquid SiO_2_, which could react with other gasses containing Si and affect the growth of the SiC NWs^39^. Therefore, alumina could play an important role in adjusting the content of the reactive silicon to promote the growth of the SiC NWs. Considering the role of alumina, an alumina-assisted VS mechanism was proposed to explain the growth of the ultralong SiC NWs obtained from the inner wall of the alumina crucible, while the model of the NWs grown on the surface of the mixture powder was controlled by the typical VS mechanism.

**Figure 6:**
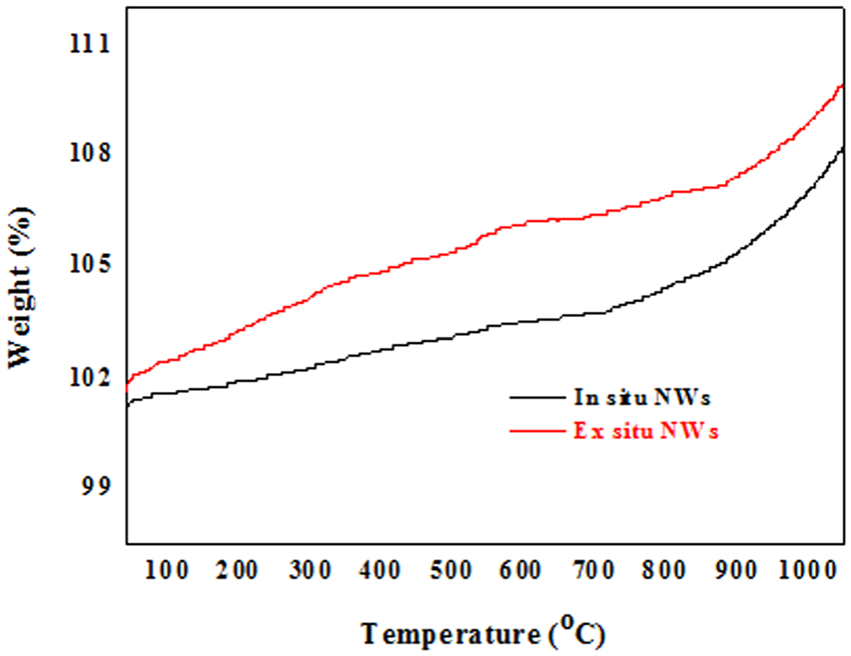
TGA curve of the SiC NWs in air at temperatures ranging from 100 °C to 1000 °C.

The thermal stability of the SiC NWs grown on the surface of the mixture powder and the inner walls of the alumina crucible in air were also investigated by thermogravimetric analysis (TGA) with a heating rate of 10 °C/min, and the TGA curve of the SiC NWs is shown in Fig. 6. Both of the TGA curve revealed a slight weight gain or loss in the SiC NWs below 1000 °C, which were ascribed to the difficulty of forming an integrated surface oxide layer prior to absolute oxidation and to the release of the moisture absorbed at room temperature. Above 1000 °C, the weight sharply increased, demonstrating that the SiC was further oxidized, which weresimilar to the results of earlier reports^54^. Moreover, the thermal stabilities of the SiC NWs obtained by this method were comparable to that of the SiC NWs^55^. It is worth noting that the thermal stability of the SiC NWs obtained from the surface of was slightly better than that of the SiC NWs obtained from the inner walls of the alumina crucible, which might be attributed to the presence of Al with a lower melting point compared to that of SiC^56^. The thermal stability in an oxidative atmosphere is of great importance for the potential applications in high-temperature environments. Therefore, the obtained SiC NWs exhibited unique optical properties and excellent thermal stability properties, which could meet the requirements of many other possible applications.

### Mechanical properties of the SiC NWs

According to the results in the previously published literature, the influence of the diameter on the mechanical properties of the NWs is very apparent^13^. Previous studies were mainly focused on the mechanical properties of SiC NWs no longer than 300 nm in diameter or SiC whiskers^10, 18–25^. In addition, the Young’s moduli of SiC NWs calculated using nanoindentation data have not been reported until now, according to our survey. Therefore, this study attempted to quantitatively measure the Young’s moduli of the SiC NWs with different diameters using Hertz’s model with *in situ* nanoindentation conducted in a hybrid SEM/SPM system^57, 58^. First, force-displacement curves were extracted to calculate the Young’s moduli of the SiC NWs with different diameters. It should be noted that the force-displacement curves obtained for the hybrid SEM/SPM system were the sum of the real penetration depth and the compressive deformation of the SiC NWs under the external force. To obtain the actual force-displacement curve, the single penetration depth (*h*) was extracted and the actual force-displacement curves were acquired after the single penetration depth (*h*) was obtained^58^. Furthermore, the actual force-displacement curves were fitted with the general Sneddon’s expression (equation (1)), in which *F* is the applied force, *h* is the penetration depth, *n* is a spherical contact equal to 1.5, and *α* is an unknown constant that was calculated from a power-law fit^58^. Meanwhile, reference materials were utilized to estimate the tip radius using equation (2), where *α*_*ref*_ and *E*_*r*(*ref*)_ are the fitted parameter and reduced elastic modulus of the reference sample (a silicon wafer), respectively, and the reduced elastic modulus of the test sample (*E*_*r*_) was calculated using equation (3)^51^. The Young’s modulus of the sample (*E*_*s*_) could be obtained using equation (4), where the subscripts *s* and *I* denote the sample and tip, respectively. *ν* is Poisson’s ratio, and *E* is Young’s modulus^58^.

1 $$F={\rm{\alpha }}{h}^{n}$$

2 $$R={(\frac{3{{\rm{\alpha }}}_{{\rm{ref}}}}{4{E}_{r({\rm{ref}})}})}^{2}$$

3 $${E}_{r}=\frac{3\alpha }{4\sqrt{R}}$$

4 $$\frac{1}{{E}_{{\rm{r}}}}=\frac{(1-{v}_{{\rm{s}}}^{2})}{{E}_{{\rm{s}}}}+\frac{(1-{v}_{{\rm{I}}}^{2})}{{E}_{{\rm{I}}}}$$

**Figure 7:**
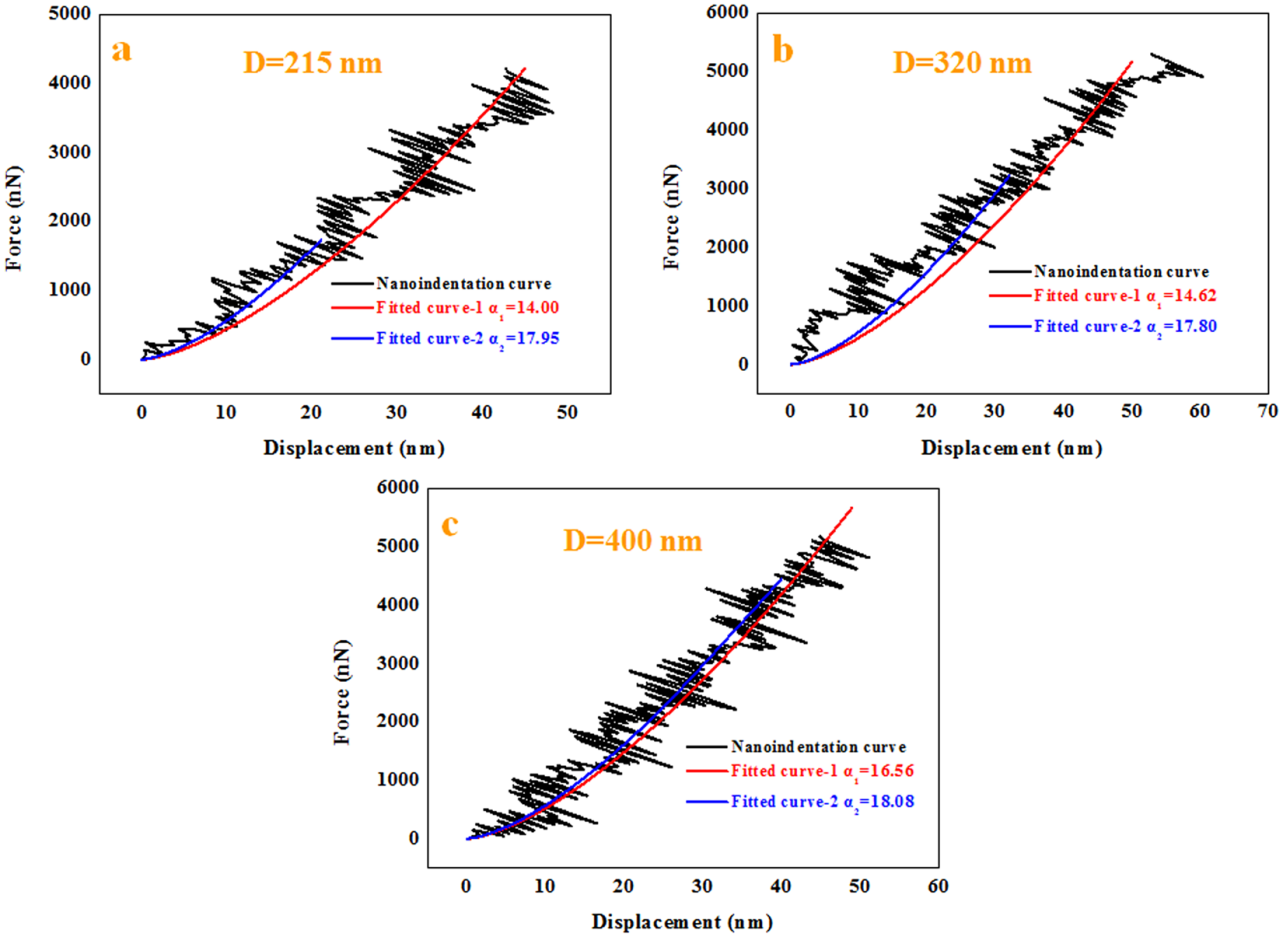
Force-displacement curves for different diameters of the samples extracted from nanoindentation experiments with different fitted curves as the displacement increased. The diameters were (**a**) 215 nm, (**b**) 320 nm and (**c**) 400 nm.

**Figure 8:**
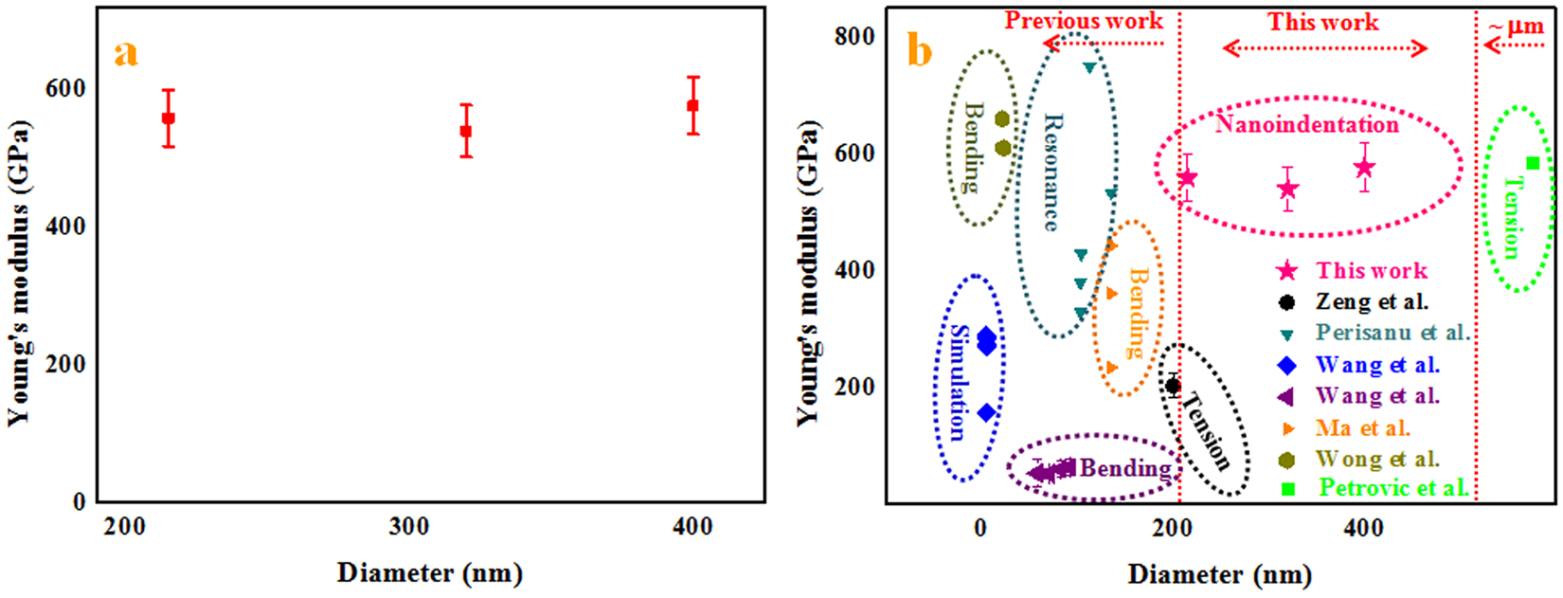
(**a**) The Young’s modulus values as a function of the diameter of the SiC NWs. (**b**) The Young’s modulus of the SiC NWs and whiskers as a function of the diameter.

The calculation procedure described above could be used to calculate the Young’s moduli of the SiC NWs grown on the inner wall of the alumina crucible. According to the obtained force-penetration depth curves, the Young’s moduli of the SiC NWs with different diameters were calculated using the above equations. Typical curves are shown in Fig. 7. To study the effect of the diameter on the Young’s moduli of the SiC NWs, three different diameters of NWs were used, including approximately 215 nm, 320 nm and 400 nm. The loading curves were fit to the force-displacement curves to calculate the Young’s moduli of the SiC NWs. A general rule for the nanoindentation measurements should be noted: the effective indentation depth should be deep enough to minimize the effect of the surface, while the depth of the indentation should also be less than 10% of the sample thickness when the sample is mounted on another substance, which can lead to differences between the measured values due to the effects of the substrate^59^. Therefore, the effective displacements of the penetration depths for the different SiC NW diameters should have been less than 21.5 nm, 32 nm and 40 nm. Meanwhile, owing to the limitation of the effective size of the probe, the effective displacements of the penetration depths of the different SiC NW diameters were selected to be 21 nm, 32 nm and 40 nm. From Fig. 7, it can be seen that the values of *α* calculated from a power-law fit changed with the displacement. The values of *α* decreased when the displacement increased due to the lower Young’s moduli of the silicon wafer compared to the Young’s moduli of the SiC NWs. Furthermore, there was an obvious downward trend in the force-penetration depth curve, leading to a lower value of *α*, as shown in Fig. 7. According to the effective values of *α* (17.95, 17.80 and 18.08) of the different SiC NW diameters and the above equations, the Young’s moduli of the SiC NWs were approximately 559.1 GPa, 540.0 GPa and 576.5 GPa, respectively, using the fitted parameter *α*_*ref*_ and the reduced elastic modulus *E*_*r*(*ref*)_ for the reference sample (silicon wafer) of 23.075 and 178.3 GPa, respectively, the properties of the silicon indenter tip (*ν*_*I*_ = 0.27 and *E*_*I*_ = 169 GPa) and an *ν*_*s*_ value of 0.19 for the SiC NWs^1, 60^. The apparent Young’s moduli of the SiC NWs were independent of the diameters of the NWs, as shown in Fig. 8a.

Figure 8b shows the measured Young’s moduli of the SiC NWs as a function of the NW diameter^10, 18–25^. Until now, many methods have been used to test the Young’s moduli of SiC NWs and SiC whiskers, and the Young’s moduli have also not been clearly size dependent. The highest Young’s moduli reported in the previous literature was approximately 750 GPa using SEM and FE of the mechanical resonances of single-clamped, batch-fabricated SiC nanowires as well as an extensive theoretical description^19^. Meanwhile, Lieber and co-workers reported the first measurements of the Young’s moduli of SiC NWs using AFM based bending tests, and the Young’s modulus of the SiC NWs with a 21.5 nm diameter was approximately 660 GPa, which was much larger than the corresponding values of the bulk SiC and SiC whiskers^18^. From Fig. 8b, the collective data for whiskers and NWs showed no clear size effects on the Young’s modulus, which agreed well with the results reported by Makeev *et al*. who found that the moduli of SiC NWs are independent of the wire diameter during axial loading^10, 61^. The measured Young’s moduli were scattered with an average value of 558.5 GPa and the average value was within the range for bulk SiC (503–600 GPa)^10^. Compared to the results in previous literature, these experimental data covered a wider range of NW diameters (215–400 nm), to the best of our knowledge, and thus, the evaluated mechanical properties of NW for a wide range of diameters are valuable for a better understanding of NW mechanics.

However, it is worth noting that although no clear size effect on the Young’s modulus was observed, the values of the Young’s moduli obtained by this approach were comparable to or larger than those obtained from many other methods. For this deviation in the values of the Young’s moduli, the reason can be concluded by the following uncertainties: (i) the boundary and loading conditions, (ii) the diameter or cross-sectional area of the NW, (iii) the way of the sample was fixed or handled, and (iv) other factors, including impurities and defects^62^. In addition, the surface effects cannot be ignored due to the effective displacements of the penetration depth according to the previous literature^61^. From a general perspective, the diameters of the SiC NWs were approximately 215 nm, 320 nm and 480 nm, which did not cause obvious surface effects on the Young’s moduli of the SiC NWs. However, the effective displacements of the penetration depths of the tested NWs were only approximately 21 nm, 32 nm and 40 nm, respectively, and thus, the surface effects could not be ignored, which could lead to higher Young’s modulus values. Compared with the original size of the NW, the effective displacements of the penetration depth of the tested NWs were all below 50 nm, and the Young’s moduli of the NWs were expected to increase with the decreasing crystal size. Meanwhile, although there were some defects in the NWs, according to the characterization results, it should be noted that only a point contact was used along the radial direction during the nanoindentation measurement, suggesting that the defects might not have significantly affected the Young’s moduli compared to the other tests, including the *in situ* tension tests^63^. In addition, because no pretreatment was employed before the nanoindentation measurement, the Young’s moduli obtained with this method might have been larger than the values obtained from other methods. For example, the samples for the tension tests were cut with a focused ion beam method and fixed to the tip and cantilever by electron beam-induced deposition, which could generate surface damage to lower the value of the Young’s modulus similar to the results reported by Bei *et al*.^64^. Therefore, the comparable or even higher Young’s moduli obtained by the nanoindentation measurements could be attributed to the following reasons: the effective displacement of the penetration depth lower than 50 nm, the simple point contact, and no obvious damage to the sample when fixed or handled.

Although there were a certain deviations, the Young’s moduli of the SiC NWs obtained by different approaches provide significance guidance for the application of SiC NWs. For example, the Young’s moduli measured by nanoindentation can provide guidance for applications with compression involved, and the values obtained by the *in situ* tension tests provide guidance for applications related to deformation in the axial direction. In addition, these findings are a progression toward the complete understanding of SiC NW mechanics. Meanwhile, additional theoretical studies and further experiments are underway to understand the origins of the size (including their diameter and length) dependence of the mechanical properties of SiC NWs.

## Conclusions

The optical properties, thermal stability and nanomechanical properties of ultralong SiC NWs were investigated, which were prepared using an efficient method with silicon and phenolic resin. Different from other previous results, the synthesized SiC NWs exhibited unique optical properties, with visible luminescence and two obvious emissions located at 527.8 nm (2.35 eV) and 438.9 nm (2.83 eV), and excellent thermal stability up to 1000 °C with a slow sample weight increase. The values of the Young’s moduli of the SiC NWs were found to be independent of the diameters of the SiC NWs, and the corresponding Young’s moduli of the SiC NWs were approximately 559.1 GPa, 540.0 GPa and 576.5 GPa for the diameters of 215 nm, 320 nm, and 400 nm, respectively. This approach not only offers an effective means of preparing ultralong SiC NWs on an industrial scale but also provides value and guidance for studying and understanding the properties of SiC nanomaterials to expand their possible applications.

## Methods

### Materials

Commercially available raw materials were used to prepare the ultralong SiC nanowires described in this work. Commercial phenolic resin and silicon (Si, 100 nm, Hefei Kaier Nanometer Energy & Technology Co., Ltd, China) were used as the raw materials.

### Preparation of the ultralong SiC NWs

The detailed synthesis process can be found elsewhere, which is described briefly, as follows. The weight ratio of phenolic resin to silicon was 3:1, and the mixture powder was homogeneously mixed using a high-frequency mixer for 30 min and placed in a ceramic crucible, and then the crucible was sent into a tube corundum furnace. High-purity argon gas (99.999%) with a flow rate of 50 ml/min was introduced into the furnace and kept flowing during the experimental process. The furnace was heated to 1450 °C with a slow heating rate, and the temperature was maintained for 2 h and cooled to 500 °C also with a slow cooling rate. Then, the samples were naturally cooled to room temperature. Finally, white, wool-like products, namely, SiC NWs were obtained on the surface of the mixture powder and the inner wall of the ceramic crucible.

### Characterization of the composition, morphology and microstructure of the SiC NWs

X-ray powder diffraction (XRD, X’PERT PRO MPD, Holland), scanning electron microscopy (SEM, HELIOS NanoLab 600i, USA) equipped with elemental area scanning, transmission electron microscopy and high-resolution transmission electron microscopy (TEM and HRTEM, Tecnai G^2^-F30, USA) were employed to analyze the composition, morphology and microstructure of the as-obtained SiC NWs.

### Characterization of the properties of the SiC NWs

The thermal stability of the SiC NWs was characterized using thermogravimetric analyses (TGA, TA Instruments TGA 2050, USA) at a rate of 10 °C/min in air. Photoluminescence (PL) measurements of the SiC NWs were carried out in an ultraviolet-visible spectrophotometer (Labram HR800) with a 325 nm He-Cd laser as the excitation source. *In situ* nanoindentation experiments were conducted using a hybrid SEM/SPM system, which depended on high-magnification SEM as a visual feedback system to indent the NWs accurately with a cantilever probe. A more detailed description of the experimental setup can be found in previous literature.

## Electronic supplementary material

Supplementary information
