# Supplementary material for: Synthesis and characterization of ultralong SiC nanowires with unique optical properties, excellent thermal stability and flexible nanomechanical properties
Source: Sci Rep. 2017 Jun 7;7:3011. doi: 10.1038/s41598-017-03588-x (PMC5462820; doi:10.1038/s41598-017-03588-x)
Supplement: Supplementary file 1 — Supplementary information [file 41598_2017_3588_MOESM1_ESM.pdf]

Synthesis and characterization of ultralong SiC nanowires with unique optical properties, excellent thermal stability and flexible nanomechanical properties

Ping Hu[1], *Shun Dong[1], *Xinghong Zhang[1], Kaixuan Gui[1], Guiqing Chen[1], Ze Hu[2]

[1]Science and Technology on Advanced Composites in Special Environment Laboratory, Harbin Institute of Technology, Harbin 150001, PR China

[2] School of Computer Science and Technology, Harbin Institute of Technology, Harbin 150001, PR China

[*]*Corresponding author*: Tel/fax: +86 451 86403016.

E-mail address: dongshunhit88@163.com (S.Dong);zhangxh@hit.edu.cn (X.H. Zhang)

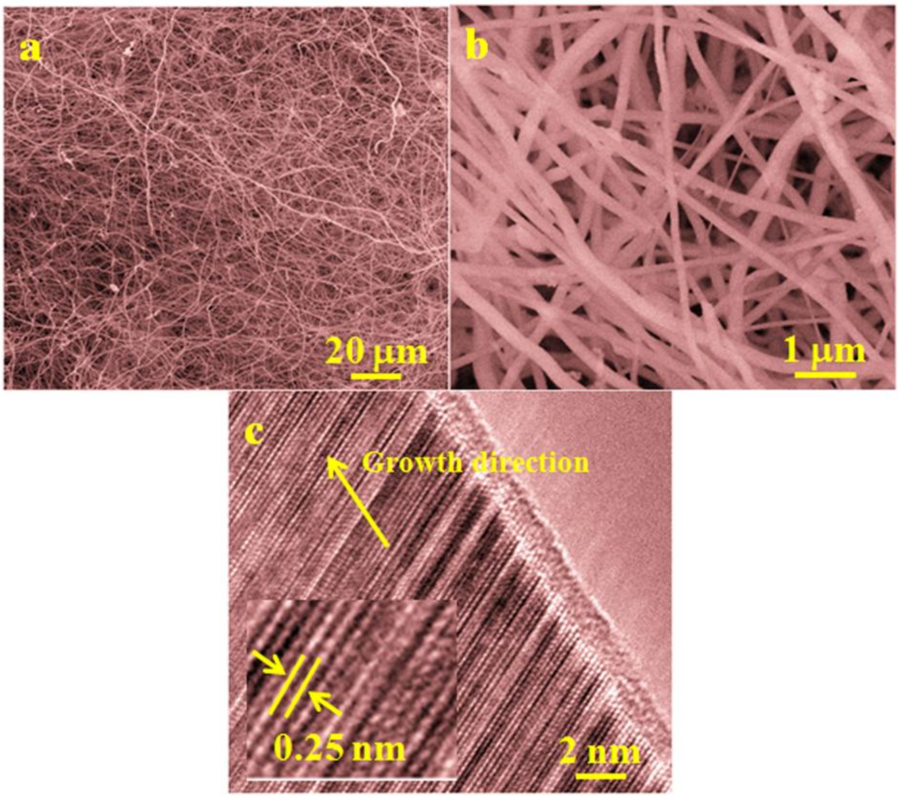

Fig. S1 (a)-(b) SEM and (c)-(d) TEM images of white, wool-like, products grown on the surface of the mixture powder. The inset image in (c) shows the partial enlarged image of (c).

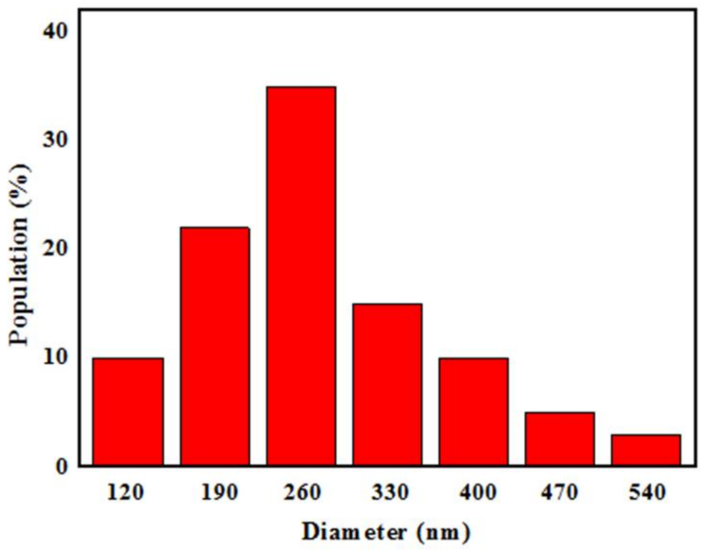

Fig. S2 The width distribution histogram of the white, wool-like, products grown on the surface of the mixture powder.

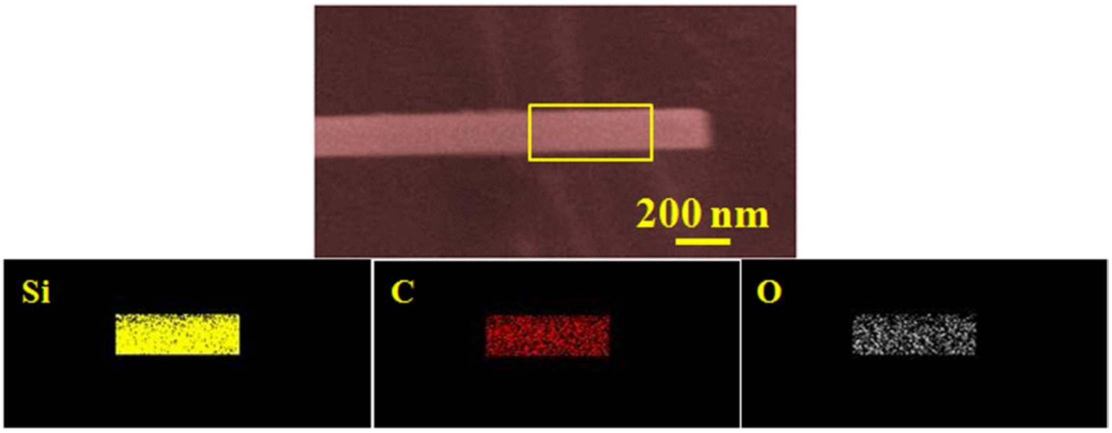

Fig. S3 Elemental area scans of the single SiC NW grown on the surface of the mixture powder.
